# Where do Protein Bodies of Cereal Seeds Come From?

**DOI:** 10.3389/fpls.2016.01139

**Published:** 2016-08-04

**Authors:** Emanuela Pedrazzini, Davide Mainieri, Claudia A. Marrano, Alessandro Vitale

**Affiliations:** Istituto di Biologia e Biotecnologia Agraria, Consiglio Nazionale delle RicercheMilano, Italy

**Keywords:** cereals, evolution of subcellular compartments, prolamins, protein bodies, protein sorting, seeds

## Abstract

Protein bodies of cereal seeds consist of ordered, largely insoluble heteropolymers formed by prolamin storage proteins within the endoplasmic reticulum (ER) of developing endosperm cells. Often these structures are permanently unable to traffic along the secretory pathway, thus representing a unique example for the use of the ER as a protein storage compartment. In recent years, marked progress has been made in understanding what is needed to make a protein body and in formulating hypotheses on how protein body formation might have evolved as an efficient mechanism to store large amounts of protein during seed development, as opposed to the much more common system of seed storage protein accumulation in vacuoles. The major key evolutionary events that have generated prolamins appear to have been insertions or deletions that have disrupted the conformation of the eight-cysteine motif, a protein folding motif common to many proteins with different functions and locations along the secretory pathway, and, alternatively, the fusion between the eight-cysteine motif and domains containing additional cysteine residues.

## Introduction

Almost 50% of the global food protein supply comes from cereal seeds (faostat.fao.org). Most of these proteins accumulate in protein bodies (PB), large polymers formed in the endoplasmic reticulum (ER). First discovered in the endosperm cells of maize developing seeds (Duvick, 1961), PBs are the most striking example for an alternative, perhaps surprising, use of the ER.

The ER is the port of entry of the secretory pathway, which leads to the cell surface or the inner hydrolytic compartments using the Golgi apparatus and various endosomes as intermediate sorting stations. The ER takes care of promoting efficient, correct structural maturation of proteins that enter the secretory pathway and selects for disposal those with incurable structural defects (Anelli and Sitia, 2008). In this respect, it is therefore mainly a compartment of transit. Its use to temporarily store proteins is very uncommon and almost exclusive to plants (Herman and Schmidt, 2004). More strikingly, the permanent ER residence of proteins that do not play a role in typical ER functions seems a feature unique to the major storage proteins of *Poaceae* seeds, the most studied being those of cereals (Shewry and Halford, 2002).

## ER-Located Protein Bodies Evolved with Grasses

The *Poaceae* family (commonly termed grasses) originated less than 80 million years ago (Gaut, 2002), a long time after the most common classes of seed storage proteins, the 2S albumins and the 7S/11S globulins universally present in seeds and even in fern spores, had appeared (Rödin and Rask, 1990; Shutov et al., 1998). 2S albumins and 7S/11S globulins accumulate in protein storage vacuoles (PSVs, Vitale and Hinz, 2005), which are therefore the first subcellular compartment that evolved to store seed proteins. PSV proteins start their life in the ER and reach their destination mainly by Golgi- and endosome-mediated traffic, even if direct traffic from the ER to PSVs also occurs (Vitale and Hinz, 2005). PSVs have higher luminal pH and lower hydrolytic activity than vegetative, lytic vacuoles and they are ready to receive hydrolytic enzymes during germination, when they are transformed into lytic vacuoles for the rapid degradation of storage proteins.

Prolamins evolved with grasses, where they are in most cases the major, or almost exclusive, storage proteins (Shewry and Halford, 2002). Unlike the soluble 2S albumins and 7S/11S globulins, most prolamins rapidly form very large polymers that become insoluble to various extents because of inter-chain disulfide bonds or hydrophobic interactions. These electron dense, round-shaped structures with diameters between 0.5 and 2 μm were originally termed protein granules (Duvick, 1961; Khoo and Wolf, 1970) and then renamed PB (Graham et al., 1962). PB often detach from the ER cisternae, but retain ribosomes on their cytosolic face and do not fuse with other subcellular structures (Burr and Burr, 1976; Yamagata and Tanaka, 1986). Rice, maize, and sorghum prolamins exclusively form PB, whereas other prolamins, such as those of wheat, can either form PB or traffic through the Golgi complex to PSVs, or undergo autophagy (Rubin et al., 1992). The destiny depends on the individual prolamin, but also on the seed developmental stage: in wheat, the ability to traffic is more prominent at early stages and the formation of PB in the ER lumen favored at later stages (Tosi et al., 2009).

## A Wide Superfamily of Small Cys-Rich Proteins

Common features of many, but not all, prolamins (**Table 1**) are a repeated domain rich in Pro or Gln residues (hence the name) and a domain derived from the eight-cysteine motif (8CM, José-Estanyol et al., 2004), characterized by eight Cys residues arranged in a specific order, forming four intra-chain disulfide bridges necessary to maintain a scaffold of alpha-helical segments connected by variable loops. The 8CM motif is common to a wide group of proteins termed the prolamin superfamily, with different functions and localizations along the secretory pathway. Besides many prolamins, the superfamily includes cereal α-globulins, trypsin inhibitors and α-amylase inhibitors, as well as hybrid proline-rich proteins (HyPRPs), non-specific lipid transfer proteins (nsLTPs) and 2S albumins, the latter three classes being widespread in plants. HyPRPs are cell wall proteins. nsLTPs are also secreted, often remaining anchored to the plasma membrane via a GPI anchor. 2S albumins, cereal α-globulins, trypsin inhibitors and α-amylase inhibitors are seed proteins accumulated in PSV. In prolamins and in the PSV-located members of the group three conserved regions, termed A, B, and C, can be identified within the 8CM motif, which in this case is therefore also termed ABC domain (Kreis et al., 1985).

**Table 1:** Summary of the main characteristics of the rice, maize, and wheat prolamins of group III and II (Xu and Messing, 2009) analyzed in this review.

Plant	Prolamin group	Prolamin	Repeated domain	Extra cysteine residues	8CM	Requiring reducing agents to be solubilized	PB biogenesis in heterologous systems
Rice	II	10 kD oryzeins	NO	YES	Altered by deletions	n.d.	n.d.
	II	16 kD oryzeins	NO	YES	Altered by deletions	n.d.	n.d.
	II	13 kD oryzeins	NO	NO	Altered by deletions	YES	YES (13a polypeptide)
Maize	II	β-zein	NO	NO	Altered by deletions	YES	YES
	II	27 kD γ-zein	Medium	YES	Intact	YES	YES
Wheat	II	γ-gliadin	Medium	NO	Intact	NO	Dubious
	III	HMW glutenins	Long	NO	Altered by insertion of the long repetitive domain and deletions	YES	YES


*n.d.: left determined.*

Based on the presence or not of the 8CM/ABC domain and additional Cys residues available for inter-chain disulfide bonds, prolamins have been divided into high molecular weight (HMW), cysteine rich (S-rich), cysteine poor (S-poor), and α-prolamins, the latter being the only group that does not seem related to 8CM proteins (Shewry and Halford, 2002). Based on amino acid sequence similarity and phylogenetic analysis, prolamins have also been divided into three groups named I, II, and III (Xu and Messing, 2009). The two criteria indeed largely overlap: α–prolamins are in group I, S-rich and S-poor in group II and HMW in group III. Group II members are present in all cereals. Group III is present in *Pooideae* (wheat, barley, *Brachypodium*) but not in *Panicoideae* (maize, sorghum, millet) and rice, whereas group I is only present in *Panicoideae* and perhaps *Oryzeae.*

Group III is considered the most ancient, followed by II and then I. Sequence similarity indicates that an ancestral group III prolamin may have originated by duplication of the α-globulin gene before the divergence of the different *Poaceae* subfamilies (Xu and Messing, 2009; Gu et al., 2010). Sequence alignments of orthologous regions of chromosomes suggest that the absence of group III prolamins in non-*Pooideae* families is due to gene loss after duplication (Xu and Messing, 2009).

## The Evolutionary Shift From PSV to the ER

Phylogenetic studies thus indicate that early prolamins may have evolved by insertions/mutations in a gene encoding a PSV protein, causing a tendency to shift accumulation from PSV to the ER itself. Retention in the port of entry of the secretory pathway is clearly more parsimonious than traffic to vacuoles and can therefore increase cellular fitness in tissues that spend considerable energy to accumulate storage material, but it raises two new requirements: (i) minimizing deleterious effects on ER functions, (ii) finding a new strategy for the degradation of storage proteins during germination. The rapid formation of large polymers seems to have satisfied the first requirement. For the second, a key event has probably been the change in the tissue accumulation from cotyledons to the endosperm: the latter undergoes programmed cell death at the end of maturation. The loss of membrane integrity allows access of hydrolytic enzymes synthesized and secreted by the surrounding aleurone during germination. Roles in the efficient formation of PBs are played by protein machineries specific of the endosperm (Vitale and Boston, 2008; Holding, 2014) and by elements in the coding mRNAs that target the transcripts to specific ER regions (Tian and Okita, 2014). However, a number of prolamins can efficiently form PBs also when expressed individually in vegetative tissues of transgenic plants (**Table 1**; Geli et al., 1994; Shani et al., 1994; Bagga et al., 1995; Bellucci et al., 2000; Saito et al., 2009), indicating that PB formation exploits the general folding machinery of the ER present in all tissues and that the key features to form a PB reside in prolamins themselves, as we discuss in the next two paragraphs.

## Rice and Wheat

Rice prolamins, encoded by about twenty genes, are small polypeptides (10, 13, or 16 kD) assigned to group II prolamins (Xu and Messing, 2009) and lacking any Pro- or Gln- rich repeated domain, indicating that the repetitions found in many other prolamins are not strictly necessary to form PBs (Onda and Kawagoe, 2011; Saito et al., 2012). A rice α-globulin-GFP fusion that is correctly sorted to PSV instead accumulates into ER-located PBs when the formation of the disulfide bond between Cys79 in the B region and Cys171 in the C region is inhibited by mutagenesis (Kawagoe et al., 2005). The hypervariable loop between regions B and C is much shorter in rice prolamins than in α-globulin, whereas in wheat HMW prolamins it is extensively elongated by the insertion of the very large repetitive domain rich in Pro and Gln (Kawagoe et al., 2005; Onda and Kawagoe, 2011). The authors suggested that these changes in length inhibit the formation of a critical intra-chain disulfide bond of the 8CM motif, making two Cys residues available for inter-chain disulfide bonds that allow the formation of PBs (Kawagoe et al., 2005). These insertions/deletions in α-globulin may thus have been the oldest events leading to group III formation in *Pooideae* and group II formation in *Pooideae* and rice (**Table 1**). It should also be underlined that only two 13 kD rice prolamins have the complete set of Cys of the 8CM motif and no other Cys residue, the other rice polypeptides having from 0 to 13 Cys residues in total (Onda and Kawagoe, 2011). Moreover, the two HMW prolamins of wheat (x-type and y-type HMW glutenin) have four to seven Cys residues, depending on the alleles. When expressed individually in transgenic tobacco these wheat prolamins form large polymers that are soluble only when reduced (Shani et al., 1994). Therefore, the loss of key Cys residues of the 8CM motif or the acquisition of new Cys residues can also be crucial for PB formation. Furthermore, at least once the initial rice PB core is formed, the rice prolamins without Cys residues can join it through interactions that are most probably of hydrophobic nature (Onda and Kawagoe, 2011).

Rice 13a prolamin has four Cys residues and is located in mid regions of the mature PB. When expressed in yeast as a GFP fusion, it forms insoluble PBs, whereas rice α-globulin similarly tagged with GFP is delivered to the yeast vacuole (Masumura et al., 2015). Deletions studies made on GFP fusions indicated the 13a portions corresponding to the B or C, but not A, regions form structures that resemble PBs by fluorescence microscopy, consistently with the view that the Cys residues in the B and C domains are important for PB formation. Unlike the full-length protein, these deletion mutants are soluble also in the absence of reducing agent, suggesting that there is not a direct causal relationship between insolubility and PB assembly. The polymerization state was not investigated, but the authors concluded that hydrophobic interactions may be important for the assembly of 13a prolamin into PBs (Masumura et al., 2015).

## Maize

Maize prolamins (zeins) are grouped into four classes (Holding, 2014). α-zeins, polypeptides between 19 and 22 kD encoded by about twenty genes, are the most abundant. Three genes encode the γ-zeins (16kD, 27 kD, which is another very abundant zein, and 50 kD), whereas β-zein (15 kD) and δ-zein (10 kD, 18 kD) are the products of one and two genes, respectively. During seed development, γ- and β- zeins, which belong to group II prolamins, are synthesized first. The 27 kD γ-zein and β-zein form PBs when expressed individually in transgenic vegetative tissues (**Table 1**; Geli et al., 1994; Bagga et al., 1995).

Treatments of isolated maize PBs with reducing agents solubilize the 27 and 50 kD γ-zeins but not the other zeins (Vitale et al., 1982). The 27 kD γ-zein is perhaps the best studied prolamin and plays a fundamental role in the correct formation of maize PBs. A natural duplication of its locus generates Quality Protein Maize (Wu et al., 2010; Liu et al., 2016), whereas a deletion encompassing the 27 and 50 kD γ-zein genes reduces PB number to 12% and alters PB morphology (Yuan et al., 2014). The hypothesis that the more recent group I prolamins exploited the PB forming ability of the more ancient ones is also supported by the finding that co-expression of 27 kD γ-zein enhances the accumulation of a 19 kD α-zein polypeptide expressed in transgenic tobacco (Coleman et al., 1996). 27 kD γ-zein is composed of an N-terminal domain characterized by eight repeats of the amphipathic sequence ValHisLeuProProPro and seven Cys residues, followed by a C-terminal 8CM/ABC domain. The N-terminal domain promotes PB formation when fused to other proteins that are otherwise available for intracellular traffic (Mainieri et al., 2004; Llop-Tous et al., 2010), although this dominant effect is not universal (de Virgilio et al., 2008; Ceresoli et al., 2016). Deletion of the N-terminal domain from γ-zein causes secretion of the remaining ABC domain, and the reciprocal deletion causes ER retention of the N-terminal domain, although in this case the typical round-shaped PBs are not formed (Geli et al., 1994). Zeolin, a fusion of the N-terminal domain with entire sequence of the 7S storage protein phaseolin (which does not contain Cys residues), forms PBs that, like those formed by the wild type γ-zein, are insoluble unless reduced, and it traffics along the secretory pathway when cells are treated *in vivo* with reducing agents (Mainieri et al., 2004; Pompa and Vitale, 2006). The importance of inter-chain disulfide bonds was confirmed by mutagenesis of the full-length γ-zein: when the seven Cys residues of the N-terminal domain were mutated to Ser, γ-zein was efficiently secreted (Mainieri et al., 2014). Progressive mutagenesis of these Cys residues gives rise to intermediate phenotypes, both in the full-length protein and a fusion between the N-terminal-domain and GFP: PBs become progressively smaller and ER retention is negatively affected (Llop-Tous et al., 2010; Mainieri et al., 2014). Deletion of the amphipathic repeats indicates that these are also important for PB formation (Llop-Tous et al., 2010). Altogether, these data indicate that the addition of an amphipathic sequence rich in Cys residues to an 8CM sequence has been crucial for this prolamin.

If six out of seven Cys residues of the N-terminal domain are mutated, the 27 kD γ-zein is in large part secreted, but a detectable proportion is sorted to the vacuole via a pathway that is sensitive to brefeldin A and wortmannin, two inhibitors of Golgi-mediated, vacuolar sorting (Mainieri et al., 2014). This indicates that limited multimerization avoids secretion and favors PSV sorting, whereas extensive polymerization promotes PB biogenesis. Protein sorting to PSVs requires sorting motifs recognized by specific receptors, but key recognition events could occur in the ER lumen and transient polymerization could contribute to sorting efficiency (Robinson et al., 2005; Robinson and Pimpl, 2014). Remarkably, the natural vacuolar sorting signal of phaseolin can be in part replaced by the insertion of a novel Cys residue that leads to polymerization of trimers (Pompa et al., 2010), underlining the evolutionary relationships between traffic to the vacuole and PB formation.

## A Working Model For the Origin of Protein Bodies

**FIGURE 1:**
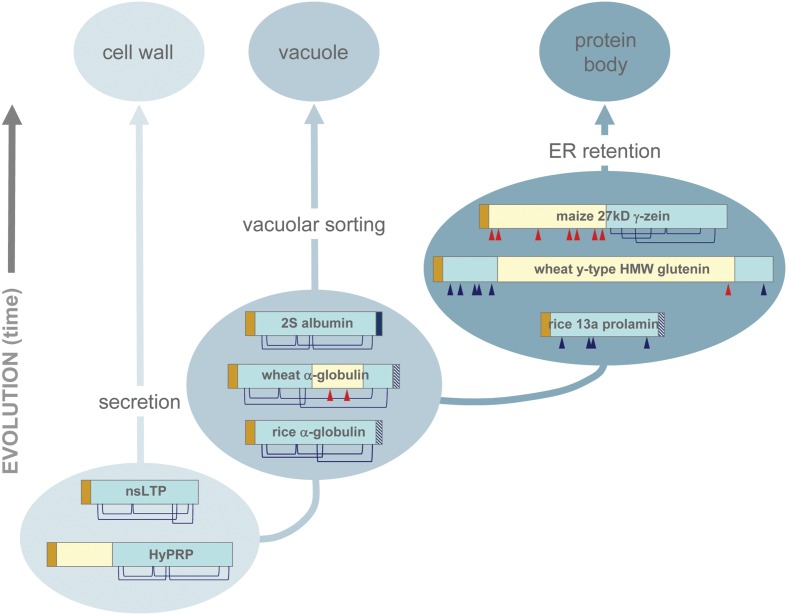
**A model of the evolutionary events at the origin of protein bodies.** The model describes the origin of prolamins that are self-sufficient for PB formation and probably have a dominant effect on those that evolved later. Proteins with 8CM motifs are secreted or sorted to the vacuole, depending on the presence or not of vacuolar sorting signals. Insertions that do not alter the disulphide bonds of the 8CM motif do not affect protein destiny. Extensive polymerization that leads to PB formation can result from insertions that inhibit the correct formation of the four intra-chain bonds, deletions that involve the loss of critical Cys residues, or additions of domains containing new Cys residues. The repetitive domains may also mediate interactions with the ER membrane. Vacuolar sorting signals may be present or absent in PB proteins, but PB formation has anyway a dominant effect on intracellular traffic. Phylogenetic analysis and the results of protein engineering and expression of individual genes indicate that the PB forming prolamins evolved from superfamily members that are sorted to the vacuole, not from secreted members. The schematic protein structures are based on the following GenBank accessions: EU968356.1 (maize nsLTP), EU964401.1 (maize HyPRP), CAA40015.1 (2S albumin), ABG68034.1 (wheat α-globulin), BAA09308.1 (rice α-globulin), 27 kD zein, ABG68035.1 (wheat y-type HMW glutenin), and BAA36697.1 (rice 13a prolamin). Brown: signal peptide; light blue: conserved 8CM motif, or portions of it; yellow: Pro-rich or Glu-rich domain; blue: vacuolar sorting sequence; striped blue: putative vacuolar sorting sequence; blue lines: intra-chain disulphide bonds of the 8CM motif; blue triangles: Cys residues originating from the 8CM motif; red triangle: Cys residues that do not originate from the 8CM motif.

The use of the ER to store proteins has evolved only recently and constitutes an interesting example of a simpler solution for protein accumulation (accumulation in the ER itself) evolving from more complex ones (sorting to vacuoles or secretion). **Figure 1** presents its possible evolutionary paths. All the evidence obtained studying prolamins that are self-sufficient to form PBs indicates that these structures have emerged by both exploiting and remodeling the 8CM scaffold. New availability of Cys residues involved in inter-chain disulfide bonds has been critical in this process and has resulted from either of two probably independent events: extensive insertions in the hypervariable region between the B and C domains characterize group III prolamins, whereas the addition of a new Cys-containing domain to an 8CM/ABC domain that can be virtually intact or mutated to variable extents characterizes many group II prolamins. The changes that have generated prolamins must thus have occurred more than once in evolution. Their success in being tolerated, without causing misfolding that would have led to degradation by ER quality control may be due to the rather simple folds of the small A, B, and C domains. The full explanation, however, must be more complex, since for example 7S globulins have a three-dimensional structure that does not tolerate great alterations (Hoffman et al., 1988; Pedrazzini et al., 1997; Lasserre et al., 2015), but zeolin is a very stable PB-forming protein fusion (Mainieri et al., 2014) and, conversely, a proportion of at least one rice 13 kD prolamin is detected in a ubiquitinated form in rice endosperm in which proteasome activity was inhibited, suggesting that normally this prolamin fails in part to fold properly and is degraded by ER quality control (Ohta and Taikawa, 2015).

It should be underlined that the formation of very large polymers is not *per se* sufficient to avoid traffic from the ER, at least in animal cells, a typical example being the secretion of procollagen fibrils (Bonfanti et al., 1998; Saito and Katada, 2015). Assembled PBs are heteropolymeric ordered structures in which certain polypeptides, such as γ- and β-zeins and rice 13–16 kD prolamins, are preferentially located at the PB surface (Lending and Larkins, 1989; Saito et al., 2012). Persistent interactions with chaperones and direct interactions with the luminal face of the ER membrane could thus play a major role in ER retention. Indeed, purified gliadins or a synthetic version of the Pro-rich repeat of 27 kD γ-zein directly interact *in vitro* with systems that mimic the inner face of the ER membrane (Kogan et al., 2004; Banc et al., 2009). It was suggested that also in gliadins the interactions could be mediated by the repeated region (Banc et al., 2009), even if the gliadin repeats are hydrophilic, unlike the amphipathic repeat of γ-zein, and the ability of γ-gliadin to form PB in the absence of other wheat prolamins is dubious (Napier et al., 1997; Tosi et al., 2009). Cell wall HyPRP proteins also have a hydrophilic, proline-rich repetitive N-terminal domain and an 8CM C-terminal domain (Dvorakova et al., 2007), but they are clearly unable to form PB. More detailed analysis and *in vivo* testing of the prolamin repetitive domains is therefore needed to determine their role in PB formation.

## Author Contributions

EP and AV wrote the manuscript. DM and CM contributed to the critical selection of the works discussed in the manuscript.

## Conflict of Interest Statement

The authors declare that the research was conducted in the absence of any commercial or financial relationships that could be construed as a potential conflict of interest.
